# No Evidence of SARS-CoV-2 Infection in Urban Wildlife of Hokkaido, Japan

**DOI:** 10.1155/2024/1204825

**Published:** 2024-08-13

**Authors:** Anastasiia Kovba, Naganori Nao, Michito Shimozuru, Mariko Sashika, Chihiro Takahata, Kei Sato, Keiya Uriu, Masami Yamanaka, Masanao Nakanishi, Genta Ito, Mebuki Ito, Miku Minamikawa, Kotaro Shimizu, Koichi Goka, Manabu Onuma, Keita Matsuno, Toshio Tsubota

**Affiliations:** ^1^ Graduate School of Veterinary Medicine Hokkaido University, Kita 18, Nishi 9, Kita-ku, Sapporo City 060-0818, Hokkaido, Japan; ^2^ Institute for Vaccine Research and Development HU-IVReD Hokkaido University, Kita 21, Nishi 11, Kita-ku, Sapporo City 001-0021, Hokkaido, Japan; ^3^ Division of International Research Promotion International Institute for Zoonosis Control Hokkaido University, Kita 20, Nishi 10, Kita-ku, Sapporo City 001-0020, Hokkaido, Japan; ^4^ One Health Research Center Hokkaido University, Kita 18, Nishi 9, Kita-ku, Sapporo City 060-0818, Hokkaido, Japan; ^5^ Faculty of Veterinary Medicine Hokkaido University, Kita 18, Nishi 9, Kita-ku, Sapporo City 060-0818, Hokkaido, Japan; ^6^ Division of Systems Virology Department of Microbiology and Immunology The Institute of Medical Science The University of Tokyo, 4-6-1 Shirokanedai, Minato-ku 108-8639, Tokyo, Japan; ^7^ Graduate School of Medicine The University of Tokyo, 7-3-1, Hongo, Bunkyo-ku 113-8654, Tokyo, Japan; ^8^ International Research Center for Infectious Diseases, 4-6-1 Shirokanedai, Minato-ku 108-8639, Tokyo, Japan; ^9^ International Vaccine Design Center The Institute of Medical Science The University of Tokyo, 4-6-1 Shirokanedai, Minato-ku 108-8639, Tokyo, Japan; ^10^ Collaboration Unit for Infection Joint Research Center for Human Retrovirus Infection Kumamoto University, Honjo 2-2-1, Chuo-ku, Kumamoto City 860-0811, Kumamoto, Japan; ^11^ Japan Science and Technology Agency CREST, 4-1-8, Honcho, Kawaguchi City 332-0012, Saitama, Japan; ^12^ Shiretoko Nature Foundation, 531 Iwaubetsu, Shari City 099-4356, Hokkaido, Japan; ^13^ School of Veterinary Medicine Hokkaido University, Kita 18, Nishi 9, Kita-ku, Sapporo City 060-0818, Hokkaido, Japan; ^14^ Division of Risk Analysis and Management International Institute for Zoonosis Control Hokkaido University, Kita 20, Nishi 10, Kita-ku, Sapporo City 001-0020, Hokkaido, Japan; ^15^ National Institute for Environmental Studies, 16-2 Onogawa, Tsukuba City 305-8506, Ibaraki, Japan; ^16^ International Collaboration Unit International Institute for Zoonosis Control Hokkaido University, Kita 20, Nishi 10, Kita-ku, Sapporo City 001-0020, Hokkaido, Japan

## Abstract

Various domestic and wildlife species have been found susceptible to and infected with SARS-CoV-2, the causative agent of COVID-19, around the globe, raising concerns about virus adaptation and transmission to new animal hosts. The virus circulation in the white-tailed deer population in North America has further called to action for virus surveillance in the wildlife. Here, we report on the first SARS-CoV-2 survey of wild animals in Japan, where frequent wildlife invasions of urban areas have occurred due to the limited predation, field abandonment, the increase of human acclimatization. Genetic testing using nasal swabs and serological screening have been conducted for sika deer, brown bears, raccoons, and raccoon dogs captured in Hokkaido prefecture from the end of the Delta variant wave to the spread of the Omicron variant, between March 2022 and February 2023. No viral RNA was detected in raccoons (0/184), sika deer (0/107), and brown bears (0/14) indicating that the virus was unlikely to spread within the population of these animal species. Among 171 raccoons, 20 raccoon dogs, 100 sika deer, and 13 brown bears, one raccoon, one brown bear, and two deer tested positive in the antibodies screening with multispecies SARS-CoV-2 N-protein ELISA. Still, ELISA-positive samples tested negative in three other serological tests, emphasizing the importance of confirming serological screening results. Our results suggested that SARS-CoV-2 was unlikely to spillback from humans to wildlife in Hokkaido during the study period, with the emergence of new variants, continuous surveillance is of utmost importance.

## 1. Introduction

The COVID-19 pandemic caused by the severe acute respiratory syndrome coronavirus 2 (SARS-CoV-2) had the devastating effects on the human health and global economy [1]. The disease was most probably a result of the virus spillover from the wild animals to humans at the Huanan seafood wholesale market [2]. The coronaviruses frequent mutations may result into the virus adaptation to the new animal hosts and raise the need for spillover monitoring [3]. The fact that the SARS-CoV-2 originated in wild animals and infected various animals other than humans has highlighted the need to understand the susceptibility of wildlife to SARS-CoV-2 and its potential impact on both animal and human health.

When animals become reservoir hosts of infection and the virus spreads among them, this could lead to the maintenance of virus variants in nature [4] or even lead to the emergence of new virus variants that may be reintroduced to the human population, being significant obstacles to managing infections in humans. In response, the Food and Agriculture Organization of the United Nations (FAO), the World Organisation for Animal Health (WOAH), and the World Health Organization (WHO) have called for surveillance of SARS-CoV-2 in wildlife [5]. Studies have identified the presence of SARS-CoV-2 in various wildlife and domesticated animal species, such as white-tailed deer (*Odocoileus virginianus*) in the US and Canada [6, 7, 8] and minks (*Neovison vison*) in the farms in Europe [6, 9] not only being significantly affected but also spreading the infection back to humans [10, 11]. Experimental infections have also shown that many animal species, including raccoons (*Procyon lotor*), raccoon dogs (*Nyctereutes procyonoides*), Norway rats (*Rattus norvegicus*), and white-tailed deer, which are commonly found in urban areas, are susceptible to SARS-CoV-2 infection [12, 13, 14, 15]. While wildlife surveillance has been conducted in various countries, including Germany [16, 17], Croatia [18], and Canada [19], the transmission of SARS-CoV-2 to wildlife in Japan remains unclear.

In Japan, the urbanization and expansion of human-dominated landscapes, increase of abandoned agricultural fields, and human behavior changes [20], have led to expansion of wild animal distributions and increase in human–wildlife interactions. Deer, bears, and other animal species invade and adapt to these human-dominated landscapes. As a result, conflicts between wildlife and people have emerged, causing economic losses, threatening local species, and posing a risk of infectious disease transmission. In Hokkaido, Japan, the frequent urban invasions of sika deer (*Cervus nippon yesoensis*), raccoons, and brown bears (*Ursus arctos yesoensis*) occur, even requiring the animal population control measures from the local government [21]. This invasion may create an optimal environment for human virus transmission to wildlife in Hokkaido. Therefore, to investigate whether wild animals, especially those living near human settlements, have been exposed to SARS-CoV-2, we conducted genetic and serological virus screenings of raccoons, sika deer, raccoon dogs (*N.p.albus*), and brown bears captured both at near urban areas and natural habitats in Hokkaido, Japan.

## 2. Materials and Methods

### 2.1. Sample Collection

Samples were collected from dead or live animals in the period between May 2022 to February 2023. Invasive raccoons were captured and euthanized for the purpose of the feral raccoons control program. Hokkaido sika deer (*C. nippon yesoensis*) were captured for population control measures, euthanized when found injured or diseased in Sapporo city or were captured as a part of continuous research of the local deer population in the Shiretoko peninsula. Samples from live Hokkaido brown bears (*U. arctos yesoensis*) were obtained during the research on the local brown bear population in Nakagawa Research Forest and Shiretoko Peninsula. Serum samples from Hokkaido raccoon dogs (*N.p.albus*), captured for the ecological research purpose, were collected from animals under anesthesia

Blood from euthanized animals was collected directly from the chest cavity or from the heart during necropsy. Sera were separated and stored at −80 or −20°C until further use. Nasopharyngeal swabs from both nostrils were collected using cotton swab sticks and placed into tubes containing viral transport medium (serum-free DMEM, 5% penicillin/streptomycin, 5% L-glutamine) or Lysing buffer (NucleoSpin® RNA Virus kit, TAKARA Bio Inc., Shiga, Japan) and transferred to −80°C until RNA extraction.

### 2.2. RNA Extraction from Swabs

Viral RNA from 200 *µ*l of swab samples was extracted using MagMAX Viral/Pathogen II Nucleic Acid Isolation Kit (Applied Biosystems, Waltham, Massachusetts, US) following the manual protocol or from 200 *µ*l of swabs immersed into Lysing buffer through the NucleoSpin RNA Virus (TAKARA Bio Inc., Shiga, Japan) according to the manufacturer's instructions. The RNA was eluted in 50 *µ*l of Elution Buffer and stored at −80°C until use.

### 2.3. SARS-CoV-2 Reverse Transcription (RT) Quantitative PCR (RT-qPCR)

RT-qPCR was performed using One Step PrimeScript III RT-qPCR Mix (TAKARA Bio Inc., Shiga, Japan) in 20 *µ*l reaction volume consisting of 10 *µ*l of One Step PrimeScript III RT-qPCR Mix, 4 *µ*l of N2 Primer/Probe mix (TAKARA Bio Inc., Shiga, Japan), 0.4 *µ*l of ROX Reference Dye, 0.6 *µ*l of RNase Free H_2_O and 5 *µ*l of sample RNA. Tenfold dilutions with the lowest detectable value of 100 copies/well of the Positive Control RNA Mix (2019-nCoV) (TAKARA Bio Inc., Shiga, Japan) were used for a copy number quantification of the virus gene.

The cycling conditions consisted of one cycle of denaturation at 25°C for 10 min and one cycle of elongation at 52°C for 2 min, followed by 45 amplification cycles of 95°C for 10 s and 60°C for 30 s. RT-qPCR was performed on the qTOWER^3^ Real-time PCR Thermal Cycler (Analytik Jena AG, Jena, Germany). All samples were run in duplicate, and samples with cycle thresholds (Ct) less than 40 in at least one replicate were considered positive.

### 2.4. Double Antigen Multispecies Enzyme-Linked Immunosorbent Assay (ELISA)

Serum samples stored were thawed on ice and tested in duplicate for anti-SARS-CoV-2 N protein antibodies with ID Screen SARS-CoV-2 Double Antigen Multispecies ELISA (Innovative Diagnostics, Grabels, France) according to the manufacturer's instruction. Briefly, a total of 25 *µ*l of dilution buffer and 25 *µ*l of each sample were added to separate wells of an ELISA microplate, and the plate was incubated at 37°C for 45 min. The wells were washed five times with wash solution, 100 *µ*l of HRP conjugate N protein recombinant antigen was added to each well, and the plate was further incubated for 30 min in the dark at room temperature. The wells were washed five times, and 100 *µ*l of substrate solution (TMB) was added to each well. The plate was then incubated in the dark at room temperature for 20 min. The reaction was stopped by adding 100 *µ*l of stop solution to each well. The optical density (OD) values of each sample were measured at 450 nm using an ELISA microplate reader (Multiskan FC, Thermo Fisher Scientific, Japan).

The sample-to-positive ratio (S/P) was calculated for each sample using the following formula: (OD sample value − OD negative control)/(OD positive control − OD negative control). Positive and negative control sera were supplied by the manufacturer. Samples with S/P values greater than or equal to 0.60 were considered positive and were further subjected to virus neutralization assay and immunofluorescence assay for antibody screening.

### 2.5. Cell Culture and Virus Neutralization Assay (VNA)

VeroE6/TMPRSS2 cells [22] were grown at 37°C in Dulbecco's Modified Eagle Medium (DMEM) (NACALAI TESQUE, Inc., Kyoto, Japan) supplemented with 10% fetal bovine serum (FCS; ICN Biomedicals Inc.), 5% penicillin/streptomycin (Gibco™, Thermo Fisher Scientific, Japan), and 5% L-glutamine (FUJIFILM Wako Chemicals, Osaka, Japan). The cells were seeded into 96-well-plates and infected with 100 50% tissue culture infectious dose (TCID_50_) of SARS-CoV-2 strains; TY/WK-521/2020 (GISAID ID: EPI_ISL_408667) and TY38-871 (GISAID ID: EPI_ISL_7571618) obtained from the National Institute of Infectious Diseases (NIID), Japan. Each ELISA-positive serum sample was serially diluted with the DMEM culture medium from fivefold to 160-fold, mixed with an equal volume of diluted virus, and incubated at 37°C for 1 hr. Cells were then washed twice and cultured with DMEM containing 2% FBS for 5 days. Every day, each well was observed for cytopathic effect (CPE) using an inverted microscope. Neutralization titers were defined as the lowest serum dilution without CPE. Serum from the SARS-CoV-2 infected hamster [23] was used as a positive control and wells without virus inoculation were used as negative controls.

### 2.6. Immunofluorescence Assay (IFA)

VeroE6/TMPRSS2 cells were cultured in 96-well-plated and infected with 100 TCID_50_ of SARS-CoV-2 TY/WK-521/2020 and TY38-871 for 1 hr. Plates were washed and cultured for 24 hr, then fixed with 4% paraformaldehyde (PFA) for 24 hr at 4°C. Fixed cells were washed three times with 1X PBS and permeabilized with 0.1% Triton X-100 (SIGMA-ALDRICH, St. Louis, Missouri, United States) in PBS at room temperature for 15 min. Cells were washed again and blocked with 2% BSA in PBS for 1 hr at room temperature followed by washing with PBS. Then, 1 : 5, 1 : 10, and 1 : 20 PBS-diluted ELISA-positive animal serum samples were added, followed by incubation at 4°C for 1 hr. SARS-CoV-2-infected hamster serum obtained in our previous study was used as a positive control [23]. For antibodies visualization, cells were incubated with PBS-diluted Protein A/G conjugated with FITC (BioVision, acquired by Abcam, Waltham, MA, USA) or anti-deer IgG (H + L) antibody, FITC-labeled (SeraCare Life Sciences, Inc., Milford, MA, USA), and 4′,6-diamidino-2-phenylindole dihydrochloride (DAPI) (Dojindo Laboratories Co., Kumamoto, Japan). After antibody staining, the cells were observed using Zeiss AXIO Vert.A1 Colibri 7 (ZEISS, Oberkochen, Germany) fluorescence microscope, and images were processed on ZEN Blue 3.4 software.

### 2.7. Neutralization Test with Lentivirus-Based Pseudovirus

In the neutralization assay, we used a lentivirus (HIV-1)-based, luciferase-expressing reporter virus pseudotyped with the SARS-CoV-2 S protein. Pseudoviruses were prepared as previously described [24, 25, 26]. Briefly, the SARS-CoV-2 S pseudoviruses (counting ~20,000 relative light units) were incubated at 37°C for 1 hr with serially diluted heat-inactivated sera (six SARS-CoV-2 N-protein multispecies ELISA positive samples) or without sera for the negative control. Later, 40 *μ*l of this mixture was added to the monolayer of HOS-ACE2/TMPRSS2 cells in white 96-well plate. At 2 days postinfection, infected cells were lysed with a One-Glo luciferase assay system (Promega, Cat# E6130), a Bright-Glo luciferase assay system (Promega, Cat# E2650), or a britelite plus Reporter Gene Assay System (PerkinElmer, Cat# 6066769). Luminescent signal was measured using a GloMax explorer multimode microplate reader 3500 (Promega) or CentroXS3 LB960 (Berthold Technologies). For each serum sample, the assays were conducted in triplicate and the 50% neutralization titer (NT50) was calculated using Prism 9 (GraphPad Software).

## 3. Results

**Figure 1:**
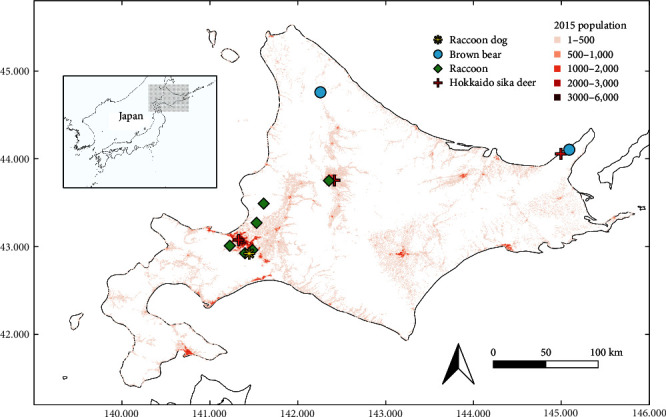
Origin of the tested samples. Map shows the sampling sites of each animal species. The red-colored mashes indicate the human population in 2015 based on the Ministry of Internal Affairs and Communications “2015 Population Census” (Data source: National Land Numerical Information Download Site, Estimated future population by 500 m mesh (2017 National Administration Bureau estimate). Accessed 13 August, 2023 at https://nlftp.mlit.go.jp/ksj/ (in Japanese)).

We collected 242 RNA samples and 251 serum samples from animals of four wildlife species in Hokkaido (Table 1). These animals were collected primarily from urban to suburban areas in central Hokkaido between May 2022 and February 2023, while for the control, some samples of deer and brown bears captured in the wild forest of Shiretoko peninsula and Nakagawa Research Forest were included (Figure 1). Since sika deer in Shiretoko Peninsula were captured alive, multiple samples were obtained from the same individual animals, resulting in 43 swab RNA samples from 32 animals and 48 serum samples from 37 animals.

The collected samples were tested for the presence of SARS-CoV-2 RNA and SARS-CoV-2-specific antibodies. We found no evidence of SARS-CoV-2 RNA in any of the tested samples. On the other hand, with the serological screening with multispecies ELISA, one raccoon sample, collected in Asahikawa, and four deer samples and one bear sample, collected in Shiretoko, tested positive (Table 1). Among those positive samples, three samples were from the same deer that had been captured in August, September, and October 2022 (Table 2). Another positive sample is from a different deer captured in September 2022. One serum sample from the raccoon captured in Asahikawa and a sample from the brown bear captured in Shiretoko in August 2022 have also been ELISA-positive (Table 2).

**Figure 2:**
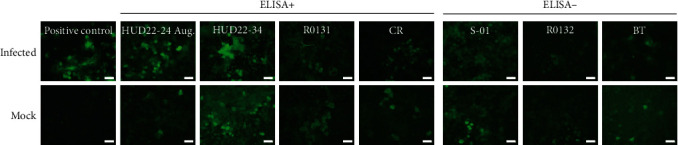
Immunofluorescence assay (IFA) results. VeroE6/TMPRSS2 cells were infected with SARS-CoV-2 TY/WK-521/2020, TY38-871, or medium (mock) for 24 hr and then fixed and incubated with the 1 : 10 PBS-diluted test serum that was positive for SARS-CoV-2 N-protein multispecies ELISA (sika deer (HUD22-24 Aug., HUD22-34), raccoon (R0131), and brown bear (CR)) or immunized hamster serum (positive control), as well as ELISA-negative representative animal serum (sika deer (S-01), raccoon (R0132), and brown bear (BT)) used as negative controls, with subsequent Protein A/G-FITC antibody staining for serum antibodies visualization. White bar = 40 *µ*m.

All six ELISA-positive samples from four animals were subjected to additional testing using a virus-neutralization assay and neutralization test with lentivirus-based pseudovirus, but no neutralizing antibodies were detected (data not shown). To further confirm the absence of anti-SARS-CoV-2 specific antibodies in the samples that tested positive in ELISA, we also conducted IFA (Figure 2). Only nonspecific staining was observed in the cells incubated with ELISA-positive deer serum (Figure 2), while clear perinucleus concentration of the virus-antibody complex has been detected in the cells incubated with SARS-CoV-2 immunized hamster serum. For deer, we also confirmed this result using antideer IgG secondary antibody (data not shown).

## 4. Discussion

We report here the first study of the SARS-CoV-2 survey in wildlife in Japan and show the absence of evidence supporting spillback events from humans to wildlife in Hokkaido. We tested mostly raccoons and deer, as only a few samples could be obtained from brown bears and raccoon dogs. SARS-CoV-2 infection experimental studies have shown the susceptibility of North American raccoons, raccoon dogs, and white-tailed deer [11, 12, 13, 14], while recent analysis also showed that raccoon dogs may have been a reservoir host for SARS-CoV-2 transmission to human in a Wuhan market in China [27], which can also explain their high susceptibility to SARS-CoV-2 [13]. Still, despite the surveillance efforts in Europe, no positive cases among raccoons [16], wild boars and red foxes [18], and raccoon dogs [17] have been confirmed so far, and our study is in line with these previous reports.

Infections and circulation of various strains of SARS-CoV-2 in wild deer, i.e., the free-ranging white-tailed deer in North America, from as early as 2020 [28], have been of utmost interest because of the potential emergence of new variants among the deer and their reintroduction to human population [4]. While Alpha, Gamma, Delta, and Omicron variants were found in the white-tailed deer in various US states [29], no positive cases were detected among white-tailed deer in Vermont, nearby area of the regions with positive animals, in 2021–2022 during Omicron spread causing unprecedented increase in human cases [30, 31]. Although SARS-CoV-2 variants show a different degree of infectivity in the laboratory [32] and wild animals [15], these results suggest that the variants spreading at the time of survey might not be a determinant of the transmission from humans to deer. So far, other than the white-trailed deer, red deer in Spain [33] and fallow deer in Spain and Ireland [33, 34], no infection has been demonstrated in wild deer at a rehabilitation center in Chile [35] and wild deer tested in Poland [36], UK [37], Germany, and Austria [38], as well as Japan (this study). Although deer species that tested negative in these studies, which belong to genera *Pudu* (*Pudu Puda*), *Capreolus* (*Capreolus capreolus*), *Cervus* (*Cervus elaphus*, *C. nippon*), *Dama* (*Dama dama*), and *Hydropotes* (*Hydropotes inermis*), are genetically distinct from the *Odocoileus* spp., which seem to be more susceptible to infection, similarity of ACE2 amino acid sequences among various deer groups suggest the susceptibility to SARS-CoV-2 infection may not be varied [39]. Moreover, cervids that were tested in Eurasia and North America belong to highly distributed species that share comparable habitats [40], suggesting the similar probability of exposure to SARS-CoV-2 infection from human population. It should be noted that the fallow deer surveyed in Ireland [34] and red deer and fallow deer surveyed in Spain [33] were captured in suburban areas where they can utilize areas that are accessible to the public, while deer in other European studies [36, 37, 38] were not accustomed to the human presence, implicating lower possibility of virus transmission. Sample size and human density at the surveillance location, as well as other factors including human behavior (deer feeding), hunting practices, intermediate hosts, and virus control measure differences, may contribute to infection transmission into the wild deer population and, therefore, should be explored further in the future studies.

Some animal species such as farm mink [10], white-tailed deer [11], cats [41], and pet hamsters [42] can become the source of human infection, and may even result in the onward transmission of SARS-CoV-2 [42]. These results indicate a high degree of virus genomic plasticity and the risk of virus adaptation to new species. Moreover, with the lifting of travel restrictions and increase of the wildlife tourism activities, the risk of wildlife infection with SARS-CoV-2 may increase. So far, only a limited number of animal species have been reported to be infected with SARS-CoV-2 in nature despite the fact that several animal species are likely to be susceptible based on the laboratory or *in silico* characterization. Taken together, the reported natural infections of SARS-CoV-2 in wild animals should be occasional events, which may depend on complicated factors, and therefore, the reports are sporadic. To identify potential reservoir hosts of the virus, continued surveillance efforts should be adapted and target highly relevant species such as various deer species and raccoon dogs. Such surveillance should also include less susceptible species given the ongoing viral mutation and the increased interaction between people and wildlife.

Interestingly, in the present study, despite the seropositivity in a few samples using multispecies SARS-CoV-2 N protein ELISA, no anti-SARS-CoV-2 antibodies have been detected in the same samples with the other tests, such as virus neutralization test, lentivirus-based pseudovirus neutralization test, and IFA, suggesting the lack of SARS-CoV-2 exposure in these animals. These results point out the importance of using a combination of serological methods of surveillance and interpreting the results appropriately due to the possible false positive results, when only one method is used.

## 5. Conclusion

During the investigation period, we did not find evidence of SARS-COV-2 transmission to wildlife in Hokkaido, Japan. As the virus spread continues and new studies indicate virus transmission to wild animals, the surveillance should also be adapted and include highly susceptible animals and animals that live in proximity to humans. Other methods of investigation such as *in vitro* use of primary cell cultures from the wildlife and analysis of genetic susceptibility of various wild animal species should also be adapted to investigate the risk of wildlife exposure to SARS-CoV-2.

## Figures and Tables

**Table 1:** SARS-CoV-2 infection in Hokkaido wildlife between May 2022 and February 2023.

Species	Test method	Number of animals (positive/tested)					
		Asahikawa	Sapporo	Shiretoko	Nakagawa	Other areas	Total
Raccoon	RT-qPCR	0/28	0/74	—	—	0/82	0/184
	ELISA	1/33	0/55	—	—	0/83	1/171

Raccoon dog	ELISA	—	—	—	—	0/20	0/20

Sika deer	RT-qPCR	0/56	0/8	0/43 ^*∗*^	—	—	0/107
	ELISA	0/47	0/5	4 ^*∗∗*^/48 ^*∗∗∗*^	—	—	4/100

Brown bear	RT-qPCR	—	—	0/9	0/5	—	0/14
	ELISA	—	—	1/8	0/5	—	1/13

^*∗*^Samples collected from 32 individual animals;  ^*∗∗*^three samples from the same animal at different time points; and  ^*∗∗∗*^samples collected from 37 individual animals.

**Table 2:** Multispecies SARS-CoV-2 N protein ELISA-positive animals.

Animal ID	Species	Sex	Capture site	Capture date	ELISA S/P (%) ^*∗*^
R0131	Raccoon	M	Asahikawa	July 1, 2022	164

HUD22-24	Sika deer	F	Shiretoko Peninsula	August 4, 2022	759
				September 29, 2022	305
				October 20, 2022	75
HUD22-34	Sika deer	F	Shiretoko Peninsula	September 29, 2022	142

CR	Brown bear	F	Shiretoko Peninsula	August 24, 2022	70

^*∗*^The average S/P (%) ratio of a duplicate measurement for each serum diluted in 1 : 2 was shown. S/P (%) > 60% is considered positive.

## Data Availability

Other information are available upon request from the corresponding authors.
